# The Validity of Google Trends Search Volumes for Behavioral Forecasting of National Suicide Rates in Ireland

**DOI:** 10.3390/ijerph16173201

**Published:** 2019-09-02

**Authors:** Joana M. Barros, Ruth Melia, Kady Francis, John Bogue, Mary O’Sullivan, Karen Young, Rebecca A. Bernert, Dietrich Rebholz-Schuhmann, Jim Duggan

**Affiliations:** 1 Insight Centre for Data Analytics, NUI Galway, H91 AEX4 Galway, Ireland; 2 School of Computer Science, National University of Ireland Galway, Galway, Ireland; 3 Psychology Department, Health Service Executive MidWest, Ennis, Ireland; 4 Psychology Department, Health Service Executive Dublin Mid Leinster, Longford, Ireland; 5 School of Psychology, National University of Ireland Galway, H91 EV56 Galway, Ireland; 6 Suicide Prevention Resource Office, Health Service Executive West, Galway, Ireland; 7 Suicide Prevention Research Laboratory, Department of Psychiatry and Behavioral Sciences, Stanford University School of Medicine, Stanford, CA 94305-5717, USA; 8 ZB MED, University of Cologne, Gleueler Str. 60, 50931 Cologne, Germany

**Keywords:** suicide, Google Trends, forecasting, autoregression, neural networks, Ireland

## Abstract

Annual suicide figures are critical in identifying trends and guiding research, yet challenges arising from significant lags in reporting can delay and complicate real-time interventions. In this paper, we utilized Google Trends search volumes for behavioral forecasting of national suicide rates in Ireland between 2004 and 2015. Official suicide rates are recorded by the Central Statistics Office in Ireland. While similar investigations using Google trends data have been carried out in other jurisdictions (e.g., United Kingdom, United Stated of America), such research had not yet been completed in Ireland. We compiled a collection of suicide- and depression-related search terms suggested by Google Trends and manually sourced from the literature. Monthly search rate terms at different lags were compared with suicide occurrences to determine the degree of correlation. Following two approaches based on vector autoregression and neural network autoregression, we achieved mean absolute error values between 4.14 and 9.61 when incorporating search query data, with the highest performance for the neural network approach. The application of this process to United Kingdom suicide and search query data showed similar results, supporting the benefit of Google Trends, neural network approach, and the applied search terms to forecast suicide risk increase. Overall, the combination of societal data and online behavior provide a good indication of societal risks; building on past research, our improvements led to robust models integrating search query and unemployment data for suicide risk forecasting in Ireland.

## 1. Introduction

Suicide is a leading cause of death and a global disease burden, accounting for nearly one million annual deaths across the world [1]. Annual suicide figures are critical to understanding risk and guiding research, including the study of biological, social, psychological, and economic factors that may vary with data monitoring [2]. However, a significant lag between monitoring and public reporting of suicides often delays and challenges real-time interventions [3]. This becomes a significant barrier when factors that affect suicide rates shift more rapidly and may have peaked and waned before their association with elevated suicide risk can be identified. The multifactorial nature of suicide risk poses a further challenge, as risk factors may change over time, according to specific demographics or subgroup types.

Suicide is the 18th leading cause of death across all ages, and the 2nd leading cause of death among young people (12 to 24 years old) [4]. In 2015, 425 deaths by suicide were recorded in Ireland, representing a rate of 9.2 per 100,000 of the population. Similar to increased risk observed in Europe and the United States [4], the majority of suicide deaths (e.g., >74%) were male [5]. The Institute of Medicine (IOM) further estimates that an additional 25 suicide attempts (100–200 for youth) occur for every suicide death, accounting for nearly 500,000 emergency visits annually in the United States [6,7]. A national suicide reduction strategy has been developed in Ireland [8], which aligns with coordinated strategies by the World Health Organization [9]. The Central Statistics Office in Ireland is responsible for releasing national suicide statistics, which are published with a delay of approximately two years or longer [5]. This issue has prompted increased calls for reporting advancements to guide epidemiology and enhanced surveillance.

Individuals at risk of suicide may use the Internet for a number of suicide-related reasons, such as to anonymously share suicidal thoughts with others and to seek out social connections [10], to access confidential support from suicide prevention service programs [11], and to visit websites that may contain information, such as on suicide methods [12]. Longitudinal studies conducted by Sueki and colleagues reported that suicide-related Internet use increased suicidal ideation and depression over time [13,14]. However, opportunities are also available to harness the positive potential the Internet offers, whereby clinicians can explore an individual’s Internet use as part of a suicide risk assessment process, as well as to develop personalized online safety practices as part of their crisis planning. Given that an estimated 85% of the global population is covered by a commercially-available wireless signal [15], and in 2012, 72% of United States Internet users searched the Internet for health topics [16], researchers have looked to Internet searches as a potentially new information source in the surveillance and monitoring of suicidal behavior to inform advancements in risk detection and intervention opportunities [3,13,14,17,18,19,20,21]. Facebook recently released a press briefing, noting real-time suicide prevention tools, which use artificial intelligence to identify signs of risk with advanced options to enhance connection to additional services (e.g., inFacebook Live), potentially providing the promise of real-time safety monitoring, as well as research that may advance risk prediction [22]. However, such approaches must be transparent and ethically sound.

Google is the most commonly-used search engine in the world, representing 74.54% of the global market share in 2017 [23]. Epidemiologists have monitored the use of Internet search engines, such as Google, to successfully track epidemics to accelerate real-time understanding of risk or data trends [24,25]. For example, by monitoring changes in help-seeking behavior in the form of Internet search volumes for phrases closely linked with a specific pathogen, disease outbreaks and epidemics can be identified and thus acted on. Google Trends is a website that acts as an online log of Internet search volumes performed on the Google.com search engine. Google Trends allows public access to statistics on queries performed on the Google search engine. It reports search volumes, as opposed to raw counts, for a particular term as a portion of the total number of searches for a given area. The data are adjusted for overall search volume in the geographical area, and the search data date back to January 2004 [26].

Several researchers have utilized Google Trends to identify outbreaks of infectious diseases, such as influenza [24,25], chickenpox [27], and gastroenteritis [28]. Ginsberg et al. [25] analyzed Google Trends search queries to track influenza-like illness in the United States. The authors reported that the relative frequency of certain queries (e.g., cold/flu remedies, influenza symptoms) was highly correlated with the percentage of physician visits in which a patient presented with influenza-like symptoms. In respect to suicide reporting, McCarthy [3] applied Google Trend analysis to the study of suicide risk on a population-wide level. Google Trends was used to generate search volumes for the terms “suicide”, “teen suicide”, “depression”, “divorce”, and “unemployment”. Google Trends data were subsequently compared to official Centre for Disease Control and Prevention statistics for the corresponding years for suicide deaths and intentional self-injury for years 2004–2007 in the United States. The results showed that, among the general population, there was no correlation between search volume for the term “suicide” and purposeful self-injury. In contrast, there was a strong negative correlation between the Internet search term “suicide” and suicide deaths (*r* = −0.9002). Importantly, data for youth (i.e., aged 18–25 years) differed markedly from those of the general population. Search volume for “suicide” was positively correlated with both intentional self-injury (*r* = 0.498) and suicide deaths (*r* = 0.699). The author hypothesized that this inverse correlation (i.e., between suicide-related Internet searches and suicides in the general population) indicates that the Internet is used by many to seek help or otherwise reduce suicide risk, which may vary significantly by age. Given that suicide-related Internet searches were positively correlated with self-injury and suicide among youth, the author proposed that this group may use the Internet to facilitate self-injury, suggesting greater Internet use risk for this demographic group.

Furthermore, several researchers have extended this research by exploring associations between Internet searches relating to suicide and suicide rates in various populations. This includes exploring the volume of search terms for suicide (e.g., “suicide”, “commit suicide”), risk factors for suicide (e.g., “depression”, “divorce”, “unemployment”), and specific suicide methods (e.g., “suicide by jumping”, “hanging”). Yang et al. [19] explored the association between monthly suicide rates in Taipei City, Taiwan, and Internet search volumes for 37 suicide-related terms during the period from January 2004 to December 2009. Results revealed that many of the Internet search terms were associated with actual suicide deaths. Searches for “major depression” and “divorce” accounted for, at most, 30.2% of the variance in suicide data. Their analysis also revealed that Internet search trends were associated with different means of suicide. Non-violent suicide was associated with searches for domestic violence and insomnia. The search trend for the title of a forbidden but popular pro-suicide book in Taiwan and Japan was associated with violent and male suicide deaths. In Japan, the monthly search volume for the terms “suicide” and “suicide method” was not significantly correlated with the monthly suicide rate. However, the volume of Google searches using the search term “utsu” (depression) was positively correlated with the suicide rates in the same or previous month and was negatively correlated with suicide rates after three months [17]. In the United States, Gunn and Lester [18] reported marginally significant positive associations between suicide rates and search volumes for the terms “commit suicide” (*p* = 0.01) and “how to suicide” (*p* = 0.07). The association between suicide rates and the search volume for “suicide prevention” was significant and positive (*p* = 0.001), suggesting that people are looking to the Internet for help and are potentially not finding it. Such findings may inform opportunities for intervention, as well as real-time monitoring of suicide risk at the population-level, in some cases, according to age and suicidal behavior.

To increase model validity, researchers have controlled for variables that may confound the relationship between suicide-related search data and suicidal behavior. Bruckner et al. [29] applied rigorous time-series routines to control for temporal patterns of suicide when exploring the association between Internet search terms and suicide rates in England and Wales from 2004–2010. The researchers also controlled for unemployment rates and Google searches in the news, which often peak after suspected suicides of celebrities, but which may or may not signal increases in help-seeking or depression. This is relevant as such cases have been previously reported in the literature and reveal the effects of media contagion [30]. For the three searches that included the term “depression”, a positive relationship with suicide in that month was found. The strongest positive relationship occurred between the Google Trends query for “depression and help” and suicide incidence in the same month (*p* = 0.002). No relationship was found between searches for “suicide” or “suicide and methods” and suicide incidence. In contrast, Kristoufek et al. [21] found that a greater number of searches for the term “depression” was related to fewer suicides, whereas a greater number of searches for the term “suicide” was related to more suicides in England between 2004 and 2013.

In 2017, Tran et al. [20] evaluated the validity and utility of Google Trends search volumes for behavioral forecasting of risk/suicide rates in the United States of America, Germany, Austria, and Switzerland. The researchers concluded that the validity of Google Trends search volumes for behavioral forecasting of national suicide rates is low, and they proposed several recommendations to increase the reliability and stability of the use of data obtained from Google Trends, which will be incorporated into the present study. Such recommendations include the use of specific search terms instead of broad terms (i.e., “suicide”), in contrast to previous approaches in the literature, and the presence/absence of quotation marks when retrieving the search query volume.

Nonetheless, the use of internet sources for statistical purposes should be used with caution. Selection bias is a predominant issue due to the uneven Internet penetration among and within countries, the population covered by these sources is also subject to daily changes, and often there is difficulty in linking the data to other datasets [31,32,33]. In detail, for search queries, one must also be wary of several factors such as changes to the search algorithm [34] and media events which lead to an unexpected behavior [35].

The present study aims to apply search query volumes to help forecast suicide outcomes in Ireland. This contrasts with the common use of historical suicide records for forecasting, without the consideration of the use of other sources. Furthermore, our comparison to the United Kingdom aims to clarify that despite cultural and geographic proximity, search behavior online can vary, thus, approaches must be targeted at country-level. This study will address a gap in the existing literature wherein the use of search query data to forecast suicide occurrences in Ireland remains unexplored. Ireland is a prime case study for the application of search query data; English is the predominant language and Internet access is present in almost 90% of the households [36]. Suicide is also a leading cause of death in Ireland, particularly in young people and women [37]. Similar research has not been conducted in this jurisdiction to date. Thus, we identify the most informative terminologies used by the population of Ireland and state the benefits of applying Google Trends for suicide forecasting in Ireland. The current study employs a broad dataset spanning eleven years. Our study requests Google Trends search volumes for search queries relating to “depression” and “suicide” and employs a collection of specific search queries (e.g., “how to commit suicide”) gathered from Tran et al. [20], in addition to suggested queries specific to Ireland. The generated search volumes are explored in terms of their relationship to Irish deaths by suicide statistics published by the Central Statistics Office, while controlling for unemployment and temporal patterns in suicide. Furthermore, we apply vector autoregression and neural network autoregression techniques to forecast the suicide outcomes in Ireland.

## 2. Materials and Methods

### 2.1. Materials

#### 2.1.1. National Suicide Records

Irish suicide deaths by occurrence on a monthly basis between January 2004 and December 2015 were provided by the Central Statistics Office (CSO) [38]. The data represents the number of suicides aggregated by the entire population (without distinguishing between age and gender), by month. The total number of reported cases between the specified period was 5938. The highest number of deaths occurred in years 2011, 2006, and 2012, with 554, 552, and 541 cases recorded, respectively. Overall, May was the month with the higher number of reported cases (588 in total), within this specific study period. The highest number of suicides reported between 2004–2015 occurred in January 2011. These data were used in our analysis of the associations between search query volumes and national suicide statistics in Ireland.

#### 2.1.2. Search Queries

In this study, we used Google Trends to retrieve time-series data for a set of search queries. This tool indicates how often a term (i.e., search query) is searched relative to its total search volume, within the user-specified time window; the calculated search rate ranges between 1 to 100. Furthermore, Google Trends can provide the values across multiple regions of the world; we exploited this feature to consider only Irish-based searches. In addition, Google Trends suggests related queries for the search terms utilized. We used this to iteratively collect related search terms which were relevant to our study, and characteristic of the Irish population. The additional queries stemmed from two generic and inclusive terms: “depression” and “suicide”. These will be referred to as root terms throughout this paper. Root terms were selected due to their relevance to suicide and replication, given their inclusion in past studies [3,18,19,20,21,39]. We also included terminology from Tran et al. [20] in our list of suicide-related terms and an additional four colloquial terms deemed relevant to depression. These terms are “top yourself” relating to suicide and “feeling down”, “got the blues”, and “baby blues” relating to depression. Due to insufficient Google Trends data, we discarded two colloquial terms and 24 other terms from Tran et al. [20]. In addition to English, other languages were considered. Given that Irish is one of Ireland’s official languages, we also utilized the root terms translated into Irish; however, because Google Trends was not capable of providing sufficient information, these terms were not applied. We also considered applying a Polish translation of the root terms given the high percentage of residents of Polish nationality living in Ireland [40]. However, due to the inconsistency of the data, this option was also discarded. All terms were searched with quotation marks indicating that, in the case of multiple words, the search must contain all terms in the stated order.

We focused on a collection period between 01/01/2004 and 31/12/2015 to match official figures provided by the Central Statistics Office. Given the variety of related queries, a manual assessment was performed to collect relevant terms only. An example of a related query to “suicide” not relevant to our study is “suicide squad”. This is the title of a film released in 2015, which does not relate to the purposes of our study and was, therefore, excluded. The list of search queries used by Tran et al. [20] and the selected queries used here are presented in Table 1. These were collected on the 30/07/2018. The data are provided on a monthly scale.

#### 2.1.3. Unemployment Records

Worldwide, past research [41,42] has shown the significant effects of unemployment on increased risk of suicide. In Ireland, the National Suicide Research Foundation (NSRF) reported that the 2008–2012 government budget policies, which were introduced during the economic crisis, had a strong negative impact on suicide and self-harm [43]. We leveraged these findings to include unemployment records, a repercussion of the fiscal measures applied in 2008–2012, as an additional source of information in the present models. Irish unemployment records from the period 2004–2015 were retrieved from the CSO [44]; the data are provided on a monthly scale.

#### 2.1.4. Data Availability

The suicide occurrences and unemployment data that support the findings of this study are provided by the CSO and are available online at https://www.cso.ie/px/pxeirestat/Statire/SelectVarVal/Define.asp?MainTable=VSD32&TabStrip=Select&PLanguage=0&FF and https://www.cso.ie/px/pxeirestat/Statire/SelectVarVal/Define.asp?maintable=MUM01&PLanguage=0. The search query search rate data that support the findings of this study are publicly available from Google Trends at https://trends.google.com/trends/.

### 2.2. Methods

#### 2.2.1. Exploratory Analysis

National suicide statistics and search query data were explored and tested using a number of methods. First, we calculated descriptive statistics to evaluate normality of the distribution of the data, according to the Jarque-Bera test [45]. To test for the presence of unit roots (i.e., a stochastic trend causing an unpredictable systematic pattern) and stationarity, we applied the Augmented Dickey-Fuller (ADF) [46] and the Kwiatkowski-Phillips-Schmidt-Shin (KPSS) [47] tests; both were applied to minimize Type I errors (i.e., rejection of the null hypothesis, in this case that a unit root is present in a time series, and that the data are stationary, respectively). In addition, to test for autocorrelation we applied the Ljung-Box test [48] with the aim of determining if the data are independently distributed, at different lags.

To measure the strength of the association between each search term and the national suicide statistics, Pearson correlation coefficients (*r*) were calculated. We used this measure to identify highly linear correlated features, which could affect the forecasting ability of our models.

#### 2.2.2. Models

Two distinct approaches were used to train the prediction models; the first approach is based on vector autoregressive models (VAR) [49] and the latter on neural network autoregression (NNAR) [50]. The VAR model is a multi-equation system where all the variables are treated as endogenous (dependent). It is defined in equation 1 where $A_{i}$ represents the coefficient matrices, a a vector of intercepts, and $\varepsilon_{t}$ the white noise.

(1) $$Y_{t}=a+A_{1}Y_{t-1}+A_{2}Y_{t-2}+\ldots +A_{p}Y_{t-p}+\varepsilon_{t}$$

The forecast of suicide occurrences is complex and past research has supported the view that single search queries volumes are not adequate to predict changes in the number of occurrences. Hence, in this research we carefully select a collection of search queries and we apply VAR allowing us to consider the model the dynamics and the interdependencies among all the time series. In addition, VAR requires a straightforward preprocessing and implementation as well as a fast training time, making it easy to use and understand by healthcare professionals.

Neural networks (NN) are a type of statistical model based on multiple nodes, organized in layers, working in parallel. The predictors form the input layer, and the forecasts form the output layer. Each node is associated with an activation function which transforms the input to the node to an output which in turn acts as an input for the nodes in subsequent layer. The input of each node is also subject to a weight, determined and updated by the learning algorithm that minimizes a cost function. The simplest NN, without hidden layers (intermediate nodes), are equivalent to linear regression; when hidden layers are present, a nonlinear function is applied to the input to the nodes. To compare the performance of linear and nonlinear models in the prediction of suicide occurrences we decided to utilize NN. These are able to model complex relationships without prior assumptions hence, not imposing any restriction on its parameters to ensure stationarity [51]. The large timespan of our data was also one of the reasons to utilize this model as it is known to require a large quantity of data. In this research, we apply the NNAR in which the lagged values of time series are used as input predictors and the output is the predicted values of the series. The model has the following mathematical representation:

(2) $$y_{t}=w_{0}+\overset{h}{\underset{j=1}{\sum }}w_{j}\cdot g\left(w_{0,j}+\overset{n}{\underset{i=1}{\sum }}w_{i,j}\cdot y_{t-i}\right)+\varepsilon_{t}$$

where $w_{ij}$ (i = 0, 1, 2, …, n; j = 1, 2, …, h) and $w_{j}$ (j = 0, 1, 2, …, h) are model parameters or connection weights; n is number of input nodes; h is number of hidden nodes; and $\varepsilon_{t}$ the white noise. In the chosen R package, the activation function is the sigmoid function and the weights of the neural network are updated using the back-propagation algorithm.

As a benchmark, we utilize an autoregressive model using only historical suicide occurrences. The search queries were then incorporated in the models for the VAR approach; for the NNAR approach, the benchmark is set by utilizing the suicide occurrences as the only data for the model training. To train the models, data corresponding to 10 years (2004–2014) were used, and for the evaluation of the forecast we utilized the data from 2015. For all models we used a monthly frequency.

#### 2.2.3. Model Evaluation

To measure the quality of our models, we used the mean absolute error (MAE), and for the selection of the most appropriate lags the Akaike Information Criterion (AIC) was deployed. The MAE represents the average of the absolute differences between predicted values and observed values; it was chosen as it provides a straightforward way of determining the prediction accuracy of our models. Lower values of MAE indicate a better fit. Regarding the AIC, given a collection of models for a dataset, it provides an estimate of the quality of each model, relative to each of the other models. The best model has the minimum AIC among all the other models.

#### 2.2.4. Software

The models and tests described in this section were applied using R 3.4.2. The following packages were used: forecast [52], vars [53], tseries [54], and dummies [55].

## 3. Results

### 3.1. Feature Selection and Normal Distribution

**Figure 1:**
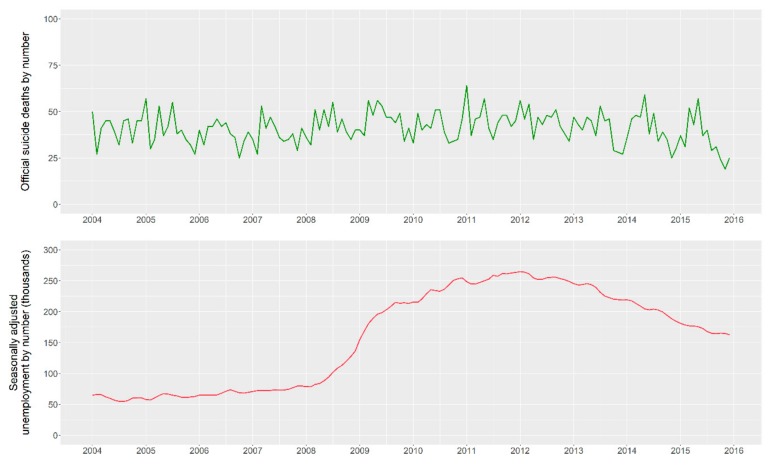
Irish official suicide statistics and Irish unemployment records. The remaining y-axis scales represent the number of occurrences. The x-axis represents the date in respect of each data point.

In total, a maximum of 32 features (i.e., 31 queries and the unemployment records) were used to obtain results. In Figure 1, we present a set of graphs which include the national suicide statistics during the period 2004–2015 and unemployment records. The figure suggests the absence of a pattern in suicide occurrences, this is verified annually as well as monthly. Regarding unemployment, rates correspondent to the period 2008–2012 show a surge in contrast to the years 2012–2015, as expected.

The results in Table 2 indicate that the distribution of official suicide deaths has negligible skewness to the left, and that it follows a platykurtic distribution, and, therefore, there is no evidence of non-normality.

### 3.2. Unit Root and Autocorrelation Assessment

Results suggest the presence of a unit root, with the exception of the search queries “depression”, “suicide methods”, “how to commit suicide”, “depression and anxiety”, “post natal depression”, “severe depression”, “baby blues”, and “suicide forum”. Given this, we elected not to transform the data through differencing, which was similar to the approach used by Kristoufek et al. [21]. The results from the Ljung-Box test at different lags suggest the absence of autocorrelations.

### 3.3. Correlation Analysis

**Figure 2:**
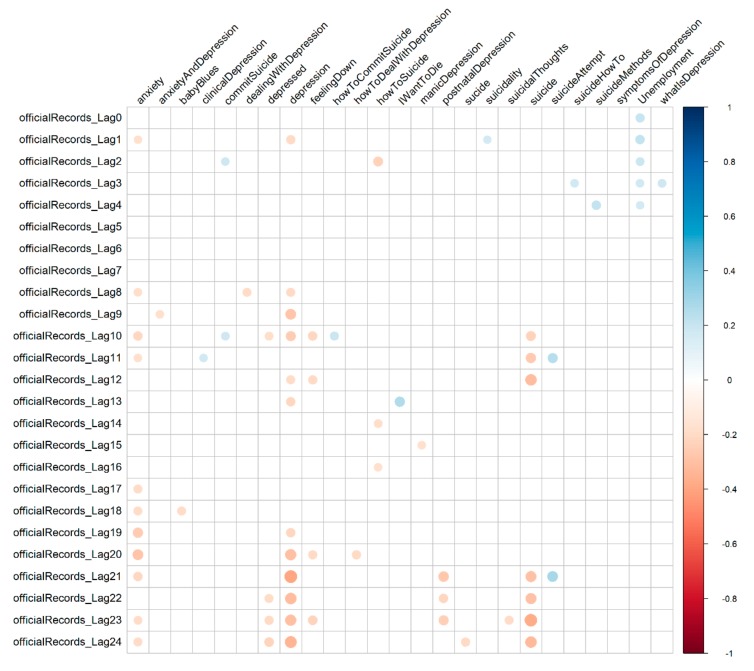
Correlation coefficients for Irish suicide data, Google search queries, and unemployment at different lags. The correlation coefficient value-color correspondence is represented on the bar on the right. Queries with a correlation not statistically significant are omitted; these include “suicidal”, “how to kill yourself”, “painless suicide”, “suicide forum”, “how to hang yourself”, “signs of depression”, “severe depression”, “post natal depression”.

Figure 2 shows the correlation of the features with the official suicide death figures at each time lag. Our analysis of correlations at different time lags suggests that positive correlations with official suicide statistics are not frequent. They were present with the queries “post natal depression”, “how to help depression”, “how to kill yourself”, “I want to die”, “suicide attempt”, “how to hang yourself”, and the unemployment records. Overall, the terms that have a higher number of correlations are “anxiety” and “depression” with 12 lags; the second root term, “suicide” shows correlations with 9 lags. The highest number of correlated queries (six), were present at lag 12 (two positive correlations with “post natal depression” and “how to kill yourself”), at lag 21 (one positive correlation with “suicide attempt”), and at lag 24 (one positive correlation with “I want to die”). Focusing on unemployment, Figure 2 shows that this is the only feature capable of having a positive correlation for multiple sequential lags, namely, lags 0 to 4. Although the behavior is similar for some search queries, these have a negative correlation with the official suicide records. This suggest that, when considering a short lag, unemployment can potentially improve forecasting models.

**Figure 3:**
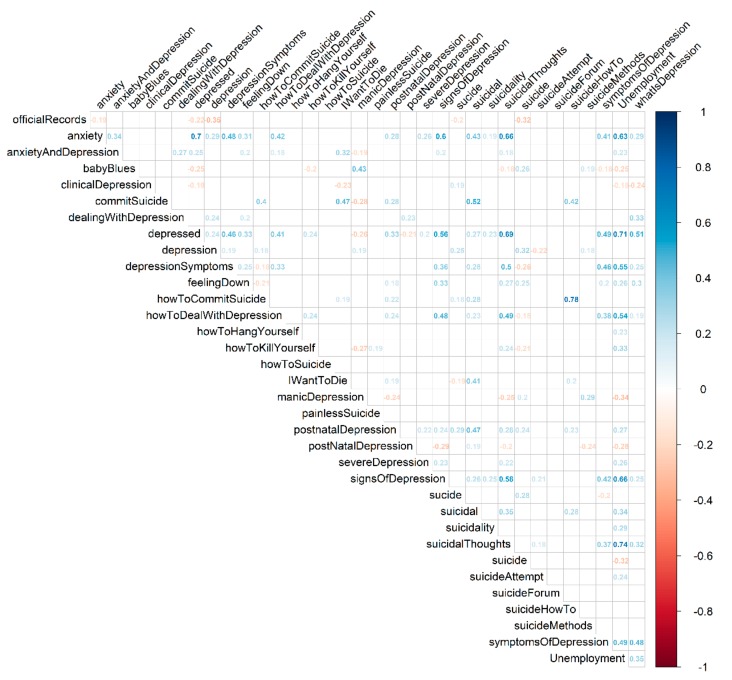
Correlation coefficients for Irish suicide data, Google search queries, and unemployment. The correlation coefficient between the 34 features and the official suicide figures are represented using a lag of 24 months. The correlation coefficient value-color correspondence is represented on the bar on the right. Cells without a color are not statistically significant.

Using a lag of 24 months—corresponding to the reporting lag in Ireland—we obtained the correlation coefficients presented in Figure 3. This correlation analysis indicates strong positive correlations between several queries. Examples include correlations between “suicidal thoughts” and unemployment (r = 0.71); “depressed” and unemployment (r = 0.71); “depressed” and “suicidal thoughts” (r = 0.65); “suicidal” and “postnatal depression” (r = 0.63); and “anxiety” and “depressed” (r = 0.61). 

### 3.4. Models

In the following models, we incorporated a combination of selected queries to identify the relationships between user-generated online content (i.e., search queries), unemployment, and official suicide statistics. Although the delay in the reporting of suicide occurrences in Ireland is 24 months, we do not have sufficient observation to use this value as a lag when including the search query volumes and unemployment records; thus, we determine the lag for the best performance for each approach. Using the AIC, we selected for the autoregressive benchmark model a lag of 2 and for the VAR Google-Unemployment model (GU) a lag of 3. The reduced model incorporates a smaller number of features considering only the search queries which contribute most to the model’s improvement. This selection was carried out by iteratively adding each feature and determining the model performance. To perform this task, we split the training data into two sets; the first includes data from 2004–2013 and it is used to train the models, the remaining data corresponding to 2014 are used for evaluation. The selected features were “depression”, and “feeling down”. The lag selected for the reduced model is 24. In addition, we incorporated the unemployment data into the reduced model to establish the impact of this variable. Table A1, available in Appendix A, summarizes the features used in each model. The results for the benchmark and VAR approach are present in Table 3; the addition of seasonal dummy variables did not provide improvements. For the benchmark, results indicate that the mean absolute error between the 2015 suicide occurrences and the model’s prediction is 10.35. When adding the two most informative features, these results decreased to 6.33. The score attained in the GU model, when using the 35 features, resulted in an MAE of 9.41. 

For the NNAR approach, the *nnetar* function from the forecast package was used to train a feed-forward neural network. An average of 20 networks are fitted for each model. For the NNAR benchmark, the determined chosen number of hidden layers is 18 and for the GU model, 30 hidden layers. In the model with the addition of data respecting the “feeling down” query 13 layers are present and when incorporating the unemployment data, the number of hidden layers is 14. Overall, the incorporation of seasonal dummy variables improved the performance; hence, we report on results also utilizing these variables.

The results in Table 4 show improvements when compared to the VAR approach with the MAE value decreasing between 0.54 and 2.19. For both approaches, the reduced model, utilizing a small number of variables, achieves the highest performance; furthermore, the presence of the shared query “feeling down” suggests its importance for the forecast of suicide occurrences. As previously indicated, the subsequent addition of more data including unemployment records does not yield improvements.

### 3.5. Misprediction Timestamp Analysis

Previous research has linked spikes in the search behavior on Google with public events [56], which suggests a potential cause for the errors in our models. Additionally, unusual events could have increased the suicide occurrences in Ireland at given times. To further explore these hypotheses, we compared our misprediction timestamps to (1) death by suicide statistics collected by the Health Service Executive (HSE), the entity responsible for health and personal social services in Ireland, and (2) news stories collected from Google News mentioning “suicide” with country specified as “Ireland”. Applying this strategy, we focused on the coinciding stated mispredictions for the reduced models from both approaches. This analysis did not contribute to the explanation of the mispredictions, given that the HSE data were scarce and Google News data provided broad results with no direct link to the Republic of Ireland.

**Figure 4:**
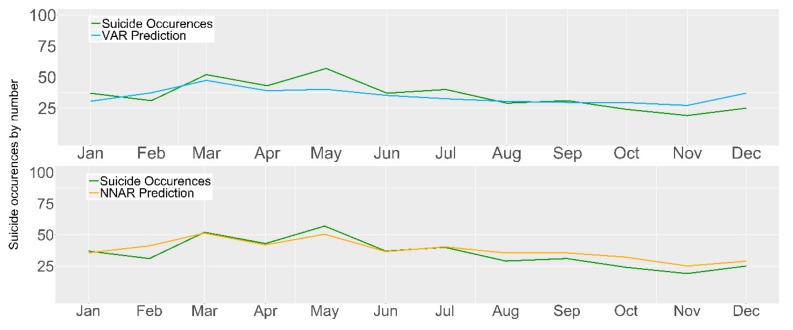
Model performance by the reduced model from the VAR and NNAR approaches for the year 2015.

In addition to the misprediction peaks (visible in Figure 4), it is also necessary to focus on the predictions below the reported number of suicide occurrences. In terms of public health policies, models with underpredictions are not as valuable as they do not alert health official of all potential future spikes in suicide occurrences (i.e., high recall), thus, it has little impact in framing new health policies. For the VAR approach, the highest prediction above the number of reported suicides (i.e., overprediction) was in May (n = 17) followed by July (n = 7). Underpredictions occur in five timestamps, namely, February (n = 6), August (n = 1), October (n = 5), November (n = 8), and December (n = 12). Regarding the NNAR approach, the highest overprediction occurs in May (n = 7). In total, six underpredictions are present with the highest occurring in February (n = 10), followed by October (n = 8), August and November (n = 6), September (n = 5), and December (n = 4).

### 3.6. Comparison with the United Kingdom 

**Figure 5:**
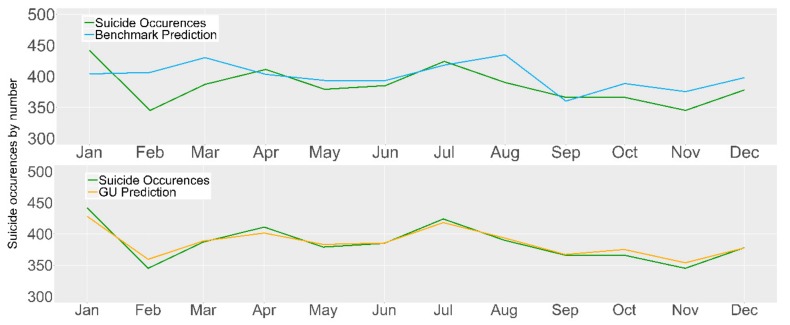
UK model performance in 2014 using the benchmark and Google + Unemployment approach.

To compare the performance of our best models (NNAR approach) with other countries we utilized the data reported by Kristoufek et al. [21], which focused on forecasting suicide occurrences in the United Kingdom (UK). The retrieved data span the period 2004–2014. Our aim was to determine the quality of the models and the chosen query terms for their forecasting capacity across different countries speaking the same language, or, conversely, to explore if these features are specifically relevant for Ireland and are not capable of a broader application. Given this, Google Trends was once again used to extract the search rate for the query “feeling down”, specifying the country as the UK. To evaluate the models, we utilize the corresponding data from January to December 2014. The results are presented in Table 5 and in Figure 5.

## 4. Discussion

### 4.1. Correlation Analysis

Contrary to what might have been expected, the results suggest that root terms do not present a high positive correlation or a higher correlation when compared to other queries. This may be explained in part by Google data not being as representative of the affected population based on official suicide figures, as was originally expected, along with such terms being too broad for the desired purpose. A detailed assessment of the suicide statistics socio-demographic information could provide more insight regarding the Google data socio-demographic representation. In addition, media events may unintentionally increase the searches for roots terms [57]. As identified previously, several terms relating to “depression” and “suicide” suggested by Google Trends were removed. These terms include examples of media events, such as “cycle against suicide” and “suicide squad”; the first refers to an awareness initiative and the latter to a film. The results could also suggest a limited use of Google by those affected by suicidal behavior, although this would appear statistically unlikely based on current suicide rates and Google user rates at population level. A competing hypothesis relates to the potential failure of the queries to represent the population’s language. For example, “baby blues” is a common colloquial term used to describe postpartum depression; however, Google query suggestions did not include such colloquial terminology. This might also be one of the reasons for the scarcity of terms highly correlated with “suicide” and “depression”.

### 4.2. Models and Comparison with the United Kingdom

Considering a lag of 24 months, corresponding to the delay in reporting suicide occurrences in Ireland, our correlation analysis results point to the effect of unemployment on users’ search behavior, particularly with terms “suicidal thoughts” and “depressed”. Furthermore, it suggests the impact of unemployment on the population and its possible influence on well-being. Past research supports the assumption that the emotional state of the population can be evaluated through people’s Internet behavior [58,59,60,61]; thus, our results further indicate one of the consequences of the harsh conditions fostered by the 2008 economic crisis in Ireland.

Overall, the addition of search queries (reduced and GU model) suggests a significant improvement, when compared to our benchmark values. In the VAR approach, the introduction of search query and unemployment data consistently performs better than the benchmark. When only utilizing the “depression” and “feeling down” queries (reduced model) we achieve the highest performance in the VAR approach, suggesting that the search volumes corresponding to these queries are able to improve the prediction of suicide occurrences in Ireland. Furthermore, the reduced models have a selected lag of 24 which coincides with the suicide reporting delay in Ireland, making this an appealing model to implement along with the current suicide prevention measures.

With the addition of unemployment data, performance did not improve considerably, which suggests that the forecasting ability of the model is mainly due to the search query data. The NNAR approach yields the overall highest performance, with the best model corresponding to the use of an additional single “feeling down”; this further supports the advantage of applying search query data, thus being an asset for suicide forecasting in Ireland. Overall, the approaches outperform traditional epidemiological methods moving on from a reliance on static risk factors to better inform policy and a population-level response.

Our findings suggest that the search behavior of users potentially exposed to or experiencing depression and/or suicidal behavior can significantly aid in the forecasting of suicide occurrences in Ireland. Models utilizing search query data improve forecasting, additionally, the query and reduced model’s performance suggests the relevancy of a limited number of queries for this task.

Although the UK data correspond to a shorter time span (i.e., 10 years) than the Irish data, the results for the forecasting of suicide occurrences improve when Google Trends data are included. Although the use of the single query “feeling down” did not yield the highest performance in the Irish data, the results suggests that regardless of the country, the search queries we selected by analyzing the literature and iterating over Google Trends suggestions are still relevant for forecasting suicide occurrences, indicating their potential generalization for countries using a common language. The lower results for the United Kingdom are potentially related to the heterogeneity of the population; Ireland as a smaller population; thus, this could have contributed to the model’s results.

Nonetheless, our findings have limitations and caveats. Google Trends provides anonymized data which restrict the analysis of age groups and socio-economic characteristics. This is important given previous research indicating stronger correlations within specific age-groups [20]. Its uneven penetration across Ireland provides difficulties in applying a finer-grain county-level geographic analysis. Furthermore, search behavior is not restricted to potential suicide occurrences, as these search queries, in particular the ones relating to “depression”, could be used by the family or friends of those experiencing a suicidal crisis. Throughout this paper, we generalize our results assuming an even use of Google Trends by the population; however, we acknowledge this as a caveat given that known inequalities in Internet access and evolving age-groups adherence are persistent influencers [62,63]. Regarding mispredictions, the reduced and Google+Unemployment models were able to consistently improve when compared to the benchmark predictions, hence delivering valuable forecasts for health officials contrasting with the current knowledge. Considering events as the possible causes of the increase in suicide occurrences or the potential increase in Google search queries, the data available for this region are not sufficiently extensive to provide a clear identification of these causes. To determine the causes of suicide increases in Ireland, a continuous collection of atypical events by national health authorities is necessary to gain a better understanding of potential precursors to such events. Future research opportunities may be provided by the implementation of a program similar to the National Violent Death Reporting System which now includes 50 states.

## 5. Conclusions

Our work extends previous research by improving the methodology, focusing on country-specific search queries, applying neural network autoregression, and applying it to the forecasting of suicide rates in Ireland, where such analysis has not been completed previously. Whilst using previously determined search queries, we extended these by gathering terms specific for the region of interest. Through a selection approach, we determined the most relevant queries which suggest the strong relevancy of pro-suicide queries (i.e., “suicide methods”, “suicidal”, “how to commit suicide”) and related medical conditions (i.e., “anxiety” and “postnatal depression”). The application of search query volume geographically restricted to Ireland shows the improvement in predicting changes in the number of suicide occurrences in the country. Furthermore, the performance achieved by the neural network autoregression suggests that this approach can yield more accurate predictions than traditional autoregression, for suicide forecasting.

Our results support the value in applying indirect sources, namely, Google Trends, for the forecasting of suicide occurrences in Ireland. These models are an added benefit for public health officials as they can anticipate changes in the number of suicide occurrences, indicating when more attention or caution should be applied. Hence, this collaborative research has created a novel tool for improving current health policies in Ireland. As suicide is influenced by a variety of psychosocial, biological, environmental, economic and cultural factors, the prediction of suicides is a highly complex task. Our approach utilizes search queries volumes and unemployment records as a proxy for some of these factors. The knowledge and applications provided by this work are three-fold: (1) this approach allows us to infer the search behavior of people at risk of suicide, i.e., the query “depressed” is commonly related to the search of “suicide”; (2) it can be used to determine early predictors of increased suicidal behavior, i.e., the search volume of suicide-related queries can indicate an increase or decrease in suicide occurrences; (3) it can provide further insights into to new trends (e.g., economical or behavioral) that are related with suicide occurrences, i.e., movie/tv releases can lead to an increase in suicide-related queries. The direct application of these findings by the public health agencies can be seen in improved and targeted suicide prevention campaigns capable of addressing the predominant issues discovered through the query analyses and to affect the largest number of people possible. For example, the search queries here utilized are suggested to be significant for the prediction of suicide occurrences; hence when these are queried, supportive messages and counselling services can be displayed to the user. Furthermore, search queries can also reveal timing and targets of prevention campaigns; as an example, highly publicized suicides (e.g., in movies, tv shows, celebrities) lead to increases in suicide related queries [64]; hence, through the identification of queries that affect suicide-related searches we can target source for an increased suicide risk.

This approach was also tested in another English-speaking county, the UK, to determine the quality and adequacy of the selected search queries for suicide forecasting. Our positive results further support the benefits of utilizing Google Trends (even in less populous countries such as Ireland), as well as the forecasting ability and generalization capabilities of a limited number of queries for suicide forecasting. Although our models were tested with United Kingdom data, other English-speaking countries, such as the United States of America, could be used for evaluations; however, it is important to acknowledge the additional challenges this brings, for example, regional and state-level differences, as well as in-state (rural and urban areas) variations.

Future research includes the identification of events that trigger increases in the public’s attention or interest in suicide, leading to a change in their online search behavior. This information could potentially be extracted from other data sources and added to the models, as additional knowledge may improve forecasting ability. Recent technological advancements show promise and new opportunities for the forecasting of suicide occurrences. Potential directions for future research include the application of machine learning algorithms, as well as natural language processing to extract information from textual records and conduct prediction with a large number of variables [65].

## Figures and Tables

**Table 1:** Search queries gathered. The first two columns indicate search queries related to “suicide” and “depression”, suggested from Google Trends. The third column represents the terms cited in Tran et al. Additional terms are present in the last column.

Suicide as Root Term	Depression as Root Term	Terms from Tran et al. [20]	Additional Terms
suicidal	anxiety	commit suicide	suicide
suicide methods	signs of depression	i want to die	depression
how to commit suicide	symptoms of depression	suicidality	baby blues
	postnatal depression	suicide attempt	feeling down
	depression and anxiety	suicide forum	
	what is depression	suicidal ideation	
	depressed	suicidal thoughts	
	post natal depression	suicide hotline	
	clinical depression	how to hang yourself	
	manic depression	how to kill yourself	
	how to help depression		
	severe depression		
	postpartum depression		
	how to deal with depression		

**Table 2:** Normality analysis results for official suicides figures. The results suggest that the data follows a normal distribution. The significance threshold used was 0.05.

Minimum	Maximum	Mean	Skewness	Excess Kurtosis	Jarque Bera	*p*-Value
19	64	41	0.02	−0.27	0.46	0.8

**Table 3:** Statistical models result for the benchmark and the VAR approach.

	AR Benchmark	Google + Unemployment	Reduced	
			“*Depression*” + “*Feeling Down*”	*“Depression*” + “*Feeling Down*” + Unemployment
MAE	10.35	9.41	6.33	9.61
Lag order	2	3	24	24

**Table 4:** Statistical models result for the NN approach.

	Benchmark	Google + Unemployment	Reduced	
			“Feeling Down”	“Feeling Down” + Unemployment
MAE	6.87	5.08	4.14	4.23
Lag order	12	12	12	12

**Table 5:** Statistical model results for the UK applying the NNAR approach.

	UK Benchmark	UK “Feeling Down”	UK Google + Unemployment
MAE	26.41	25.14	6.01
Lag Order	2	2	2

## Appendix A

**Table A1:** Features utilized in the reduced and GU models. The models also include historical suicide data.

Model	Features
Reduced	Historical suicide occurrences data , “suicide”, “depression”, “anxiety”, “suicide methods”, “suicidal”, “how to commit suicide”, and “postnatal depression”.
Google + Unemployment	Historical suicide occurrences data, “suicide”, “depression”, “suicidal”, “suicide methods”, “how to commit suicide”, “anxiety”, “postnatal depression”, “signs of depression”, “symptoms of depression”, “depression and anxiety”, “depressed”, “post natal depression”, “manic depression”, “how to help depression”, “severe depression”, “how to deal with depression”, “baby blues”, “feeling down”, “commit suicide”, “how to kill yourself”, “I want to die”, “suicidality”, “suicide attempt”, “suicide forum”, “suicidal ideation”, “suicidal thoughts”, “suicide hotline”, “how to hang yourself”, “clinical depression”, “what is depression”, and unemployment records.

## References

[1] World Health Organization (2012). Public Health Action for the Prevention of Suicide: A Framework.

[2] Parker J., Cuthbertson C., Loveridge S., Skidmore M., Dyar W. (2017). Forecasting state-level premature deaths from alcohol, drugs, and suicides using Google Trends data. J. Affect. Disord..

[3] McCarthy M.J. (2010). Internet monitoring of suicide risk in the population. J. Affect. Disord..

[4] World Health Organization Suicide Data. https://www.who.int/mental_health/prevention/suicide/suicideprevent/en/.

[5] National Suicide Research Foundation Suicide. https://www.nsrf.ie/statistics/suicide/.

[6] Goldsmith S.K., Pellmar T.C., Kleinman A.M., Bunney W.E. (2002). Reducing Suicide: A National Imperative.

[7] National Centre for Disease Control and Prevention Facts at a Glance. https://www.cdc.gov/violenceprevention/pdf/suicide-datasheet-a.pdf.

[8] Department of Health and Children (2015). Connecting for Life: Ireland’s National Strategy to Reduce Suicide 2015–2020.

[9] Fung I.C.-H., Fu K.-W., Chan C.-H., Chan B.S.B., Cheung C.-N., Abraham T., Tse Z.T.H. (2016). Social Media’s Initial Reaction to Information and Misinformation on Ebola, August 2014: Facts and Rumors. Public Health Rep..

[10] Bell J., Mok K., Gardiner E., Pirkis J. (2018). Suicide-Related Internet Use Among Suicidal Young People in the UK: Characteristics of Users, Effects of Use, and Barriers to Offline Help-Seeking. Arch. Suicide Res..

[11] Alao A.O., Soderberg M., Pohl E.L., Alao A.L. (2006). Cybersuicide: Review of the Role of the Internet on Suicide. CyberPsychol. Behav..

[12] Biddle L., Donovan J., Hawton K., Kapur N., Gunnell D. (2001). Suicide and the Internet. Psychiatr. Bull..

[13] Sueki H. (2013). The effect of suicide-related internet use on users’ mental health: A longitudinal study. Crisis.

[14] Sueki H., Yonemoto N., Takeshima T., Inagaki M. (2014). The impact of suicidality-related Internet use: A prospective large cohort study with young and middle-aged Internet users. PLoS ONE.

[15] Aggarwal N.K. (2012). Applying mobile technologies to mental health service delivery in South Asia. Asian J. Psychiatr..

[16] Fox S., Duggan M. Health Online 2013. http://www.pewinternet.org/2013/01/15/health-online-2013/.

[17] Sueki H. (2011). Does the volume of Internet searches using suicide-related search terms influence the suicide death rate: Data from 2004 to 2009 in Japan. Psychiatry Clin. Neurosci..

[18] Gunn J.F., Lester D. (2013). Using Google searches on the Internet to monitor suicidal behavior. J. Affect. Disord..

[19] Yang A.C., Tsai S.J., Huang N.E., Peng C.K. (2011). Association of Internet search trends with suicide death in Taipei City, Taiwan, 2004–2009. J. Affect. Disord..

[20] Tran U.S., Andel R., Niederkrotenthaler T., Till B., Ajdacic-Gross V., Voracek M. (2017). Low validity of Google Trends for behavioral forecasting of national suicide rates. PLoS ONE.

[21] Kristoufek L., Moat H.S., Preis T. (2016). Estimating suicide occurrence statistics using Google Trends. EPJ Data Sci..

[22] Callison-Burch V., Guadagno J., Davis A. (2017). Building a Safer Community with New Suicide Prevention Tools. Facebook Newsroom.

[23] Net Market Share Search Engine Market Share. https://www.netmarketshare.com/search-engine-market-share.

[24] Dugas A.F., Hsieh Y.H., Levin S.R., Pines J.M., Mareiniss D.P., Mohareb A., Gaydos C.A., Perl T.M., Rothman R.E. (2012). Google Flu Trends: Correlation with Emergency Department Influenza Rates and Crowding Metrics. Clin. Infect. Dis..

[25] Ginsberg J., Mohebbi M.H., Patel R.S., Brammer L., Smolinski M.S., Brilliant L. (2009). Detecting influenza epidemics using search engine query data. Nature.

[26] Boland K.M., McNutt J.G. (2013). Assessing E-Government Success Strategies using Internet Search Data. E-Government Success Factors Measures: Theories Concepts, Methodol.

[27] Bakker K.M., Martinez-Bakker M.E., Helm B., Stevenson T.J. (2016). Digital epidemiology reveals global childhood disease seasonality and the effects of immunization. Proc. Natl. Acad. Sci. USA.

[28] Pelat C., Turbelin C., Bar-Hen A., Flahault A., Valleron A.-J. (2008). More Diseases Tracked by Using Google Trends. Clin. Infect. Dis..

[29] Bruckner T.A., McClure C., Kim Y. (2014). Google Searches for Suicide and Risk of Suicide. Psychiatr. Serv..

[30] Madelyn G., Patrick J., Daniel R. (2003). Media Contagion and Suicide Among the Young. Am. Behav. Sci..

[31] Askitas N., Zimmermann K.F. (2015). The internet as a data source for advancement in social sciences. Int. J. Manpow..

[32] Citro C.F. (2014). From multiple modes for surveys to multiple data sources for estimates. Surv. Methodol..

[33] Braaksma B., Zeelenberg K. (2015). “Re-make/Re-model”: Should big data change the modelling paradigm in official statistics?. Stat. J. IAOS.

[34] Lazer D., Kennedy R., King G., Vespignani A. (2014). The parable of Google Flu: Traps in big data analysis. Science.

[35] Pack Q.R., Priya A., Lagu T.C., Pekow P.S., Rigotti N.A., Lindenauer P.K. (2017). Internet Searches for Suicide Following the Release of 13 Reasons Why. JAMA Intern. Med..

[36] CT Access and Usage by Households and Individuals. https://data.oecd.org/ict/internet-access.htm.

[37] Suicide—How Common is Suicide. https://www.hse.ie/eng/health/az/s/suicide/suicide-facts.html.

[38] Central Statistics Office VSD32: Suicide Death Rates by Sex, Year and Statistic. https://www.cso.ie/px/pxeirestat/Statire/SelectVarVal/Define.asp?MainTable=VSD32&TabStrip=Select&PLanguage=0&FF=1.

[39] Arora V.S., Stuckler D., McKee M. (2016). Tracking search engine queries for suicide in the United Kingdom, 2004–2013. Public Health.

[40] Central Statistics Office (2017). Census 2016—Summary of Results.

[41] Lewis G., Sloggett A. (1998). Suicide, deprivation, and unemployment: Record linkage study. BMJ.

[42] Blakely T.A., Collings S.C.D., Atkinson J. (2003). Unemployment and suicide. Evidence for a causal association?. J. Epidemiol. Community Health.

[43] Corcoran P., Griffin E., Arensman E., Fitzgerald A.P., Perry I.J. (2015). Impact of the economic recession and subsequent austerity on suicide and self-harm in Ireland: An interrupted time series analysis. Int. J. Epidemiol..

[44] Central Statistics Office MUM01: Seasonally Adjusted Monthly Unemployment by Age Group, Sex, Month and Statistic. https://www.cso.ie/px/pxeirestat/statire/SelectVarVal/saveselections.asp.

[45] Jarque C.M., Bera A.K. (1980). Efficient tests for normality, homoscedasticity and serial independence of regression residuals. Econ. Lett..

[46] Dickey D.A., Fuller W.A. (1979). Distribution of the estimators for autoregressive time series with a unit root. J. Am. Stat. Assoc..

[47] Kwiatkowski D., Phillips P.C.B., Schmidt P., Shin Y. (1992). Testing the null hypothesis of stationarity against the alternative of a unit root: How sure are we that economic time series have a unit root?. J. Econom..

[48] Ljung G.M., Box G.E.P. (1978). On a measure of lack of fit in time series models. Biometrika.

[49] Lütkepohl H. (2005). Periodic VAR Processes and Intervention Models.

[50] Hyndman R.J., Athanasopoulos G. (2013). Forecasting: Principles and Practice.

[51] Maleki A., Nasseri S., Aminabad M.S., Hadi M. (2018). Comparison of ARIMA and NNAR Models for Forecasting Water Treatment Plant’s Influent Characteristics. J. Civ. Eng..

[52] Hyndman R.J., Khandakar Y. (2008). Automatic time series forecasting: The forecast package for {R}. J. Stat. Softw..

[53] Pfaff B. (2008). VAR, SVAR and SVEC Models: Implementation Within R Package vars. J. Stat. Softw..

[B54-ijerph-16-03201] 54.Trapletti, A.; Hornik, K.; LeBaron, B. Tseries: Time Series Analysis and Computational Finance Description; 2018. R package version 0.10-47

[B55-ijerph-16-03201] 55.Brown, C. Dummies: Create Dummy/Indicator Variables Flexibly and Efficiently; 2012. R package version 1.5.6

[56] Fink D.S., Santaella-Tenorio J., Keyes K.M. (2018). Increase in suicides the months after the death of Robin Williams in the US. PLoS ONE.

[57] Ayers J.W., Althouse B.M., Leas E.C., Dredze M., Allem J.-P. (2016). Internet Searches for Suicide Following the Release of 13 ReasonsWhy. Implement. Sci..

[58] Woo H., Cho Y., Shim E., Lee K., Song G. (2015). Public trauma after the Sewol ferry disaster: The role of social media in understanding the public mood. Int. J. Environ. Res. Public Health.

[59] Signorini A., Segre A.M., Polgreen P.M. (2011). The Use of Twitter to Track Levels of Disease Activity and Public Concern in the U.S. during the Influenza A H1N1 Pandemic. PLoS ONE.

[60] Yang A.C., Huang N.E., Peng C.K., Tsai S.J., Bollen J., Mao H., Zeng X. (2011). Twitter mood predicts the stock market. J. Comput. Sci..

[61] Yang A.C., Huang N.E., Peng C.K., Tsai S.J. (2010). Do seasons have an influence on the incidence of depression? The use of an Internet search engine query data as a proxy of human affect. PLoS ONE.

[62] Organisation for Economic Co-operation and Development Internet Access. https://data.oecd.org/ict/internet-access.htm.

[63] Neves B.B., Fonseca J.R.S., Amaro F., Pasqualotti A. (2018). Social capital and Internet use in an age-comparative perspective with a focus on later life. PLoS ONE.

[64] Arendt F., Scherr S. (2017). The impact of a highly publicized celebrity suicide on suicide-related online information seeking. Crisis.

[65] Bernert R.A. (2018). Emerging Best Practices and Innovation in Suicide Prevention: Toward an Updated Statewide Strategic Plan for California. https://mhsoac.ca.gov/sites/default/files/documents/2018-11/Policy%20Brief_Emerging%20best%20practices%20in%20suicide%20prevention_10.17.2018.pdf.

